# Economic Evaluations of Digital Health Interventions for Children and Adolescents: Systematic Review

**DOI:** 10.2196/45958

**Published:** 2023-11-03

**Authors:** Tijana Stanic, Tuba Saygin Avsar, Manuel Gomes

**Affiliations:** 1 Department of Applied Health Research University College London London United Kingdom

**Keywords:** digital health, cost-effectiveness, economic evaluation, children, adolescents

## Abstract

**Background:**

Digital health interventions (DHIs) are defined as digital technologies such as digital health applications and information and communications technology systems (including SMS text messages) implemented to meet health objectives. DHIs implemented using various technologies, ranging from electronic medical records to videoconferencing systems and mobile apps, have experienced substantial growth and uptake in recent years. Although the clinical effectiveness of DHIs for children and adolescents has been relatively well studied, much less is known about the cost-effectiveness of these interventions.

**Objective:**

This study aimed to systematically review economic evaluations of DHIs for pediatric and adolescent populations. This study also reviewed methodological issues specific to economic evaluations of DHIs to inform future research priorities.

**Methods:**

We conducted a database search in PubMed from 2011 to 2021 using the PRISMA (Preferred Reporting Items for Systematic Reviews and Meta-Analyses) checklist. In total, 2 authors independently screened the titles and abstracts of the search results to identify studies eligible for full-text review. We generated a data abstraction procedure based on recommendations from the Panel on Cost-Effectiveness in Health and Medicine. The types of economic evaluations included in this review were cost-effectiveness analyses (costs per clinical effect), cost-benefit analyses (costs and effects expressed in monetary terms as net benefit), and cost-utility analyses (cost per quality-adjusted life year or disability-adjusted life year). Narrative analysis was used to synthesize the quantitative data because of heterogeneity across the studies. We extracted methodological issues related to study design, analysis framework, cost and outcome measurement, and methodological assumptions regarding the health economic evaluation.

**Results:**

We included 22 articles assessing the cost-effectiveness of DHI interventions for children and adolescents. Most articles (14/22, 64%) evaluated interventions delivered through web-based portals or SMS text messaging, most frequently within the health care specialties of mental health and maternal, newborn, and child health. In 82% (18/22) of the studies, DHIs were found to be cost-effective or cost saving compared with the nondigital standard of care. The key drivers of cost-effectiveness included population coverage, cost components, intervention effect size and scale-up, and study perspective. The most frequently identified methodological challenges were related to study design (17/22, 77%), costing (11/22, 50%), and economic modeling (9/22, 41%).

**Conclusions:**

This is the first systematic review of economic evaluations of DHIs targeting pediatric and adolescent populations. We found that most DHIs (18/22, 82%) for children and adolescents were cost-effective or cost saving compared with the nondigital standard of care. In addition, this review identified key methodological challenges directly related to the conduct of economic evaluations of DHIs and highlighted areas where further methodological research is required to address these challenges. These included the need for measurement of user involvement and indirect effects of DHIs and the development of children-specific, generic quality-of-life outcomes.

## Introduction

### Background

Digital health interventions (DHIs) administered through various technologies have experienced substantial growth and uptake in recent years [1,2]. DHIs are defined as digital technologies such as digital health applications and information and communications technology systems (including SMS text messages) implemented to meet health objectives [3]. DHIs can range from electronic medical records used by providers to mobile apps tailored to patients for remote monitoring, videoconferencing systems for treatment administration training, and SMS text message reminders to promote treatment adherence [4,5]. The diverse roles that DHIs can play in the health system include replacement of face-to-face meetings with health care professionals; provision of patient education and counseling services; data collection and access; health information sharing; promotion of healthy behaviors and prevention; and facilitation of patient monitoring and support through clinical examination, diagnosis, and treatment [2,6]. DHIs compete for scarce National Health Service resources (fixed budget) with other digital and nondigital technologies in the health care system. In this context of scarcity, health economic evaluations provide an assessment of the relative benefits and costs of DHIs and competing options, which is crucial evidence for informing resource allocation decisions [2]. In the context of pediatric and adolescent care settings, the most common uses of DHIs are in mental health, particularly web-based cognitive behavioral therapies (CBTs) that include both children and their families, and in weight management programs involving patient- and caregiver-reported outcomes through mobile apps [7-9].

There is an abundance of literature evaluating the clinical effectiveness of DHIs in both children and adolescents [7-9]. Several studies have found a small but substantial effect of DHIs on health outcomes in these patient groups, primarily for interventions for depression, anxiety, and weight management [7-9]. In contrast, clinical evidence on the effectiveness of DHIs and the ability to compare effectiveness across studies is limited for other diseases such as asthma, attention-deficit/hyperactivity disorder, and eating disorders [8]. This is mainly because of limitations and inconsistencies in clinical trial design, such as small sample size, variable uptake and user engagement with DHIs, lack of blinded outcome assessment, short-term follow-up, and poor specification of the extent of human support (ie, DHIs that are fully self-administered by a patient without any elements of intervention delivery or monitoring by a clinician) [8,9]. As clinical effectiveness data are essential for parameterizing cost-effectiveness models, these inconsistencies in clinical evidence can pose limitations in conducting economic evaluations of DHIs.

The current understanding of the value for money of DHIs for children and adolescents is considerably more limited. Adoption of and engagement with DHIs are likely to be strong in the pediatric and adolescent population, and hence, the potential to be effective and cost-effective (ie, through scale-up) may be high [8]. The cost-saving potential of DHIs is associated in particular with behavioral interventions for chronic conditions in this population, such as the management of obesity and anxiety through web-based CBT [10]. However, the overall cost-effectiveness of DHIs for this population is unclear as evidence seems to differ according to the clinical setting and intervention type [8,11]. For instance, web portals and telephone support programs for obesity and SMS text messaging interventions for mental health and maternal, neonatal, and child care settings are more likely to be cost-effective than telemonitoring and videoconferencing systems for posttraumatic stress disorder and cardiovascular conditions [11].

Most published economic evaluations of DHIs follow standard guidelines for the evaluation of health technologies, such as those for pharmaceutical products and interventions [2]. This includes taking a health system or payer perspective, considering only health-related benefits, and conducting cost-utility analyses (CUAs) [2]. However, economic evaluations of DHIs may raise distinct methodological issues compared with pharmaceuticals and medical devices, including the measurement of non–health care benefits and costs, user involvement, and the choice of comparator [2]. The design of economic evaluations of DHIs tailored to pediatric populations may face additional challenges as DHIs originally designed for adult populations may not offer the type of interactions, self-monitoring features, and user involvement that are appropriate to address the needs of children and adolescents [3,12]. In general, the overall methodological quality of published economic evaluations in pediatric settings is unknown, including whether economic evaluations for this population face distinct methodological issues such as the measurement of costs and effects compared with DHIs for adults.

### Objectives

To address these research gaps, we systematically reviewed economic evaluations of DHIs targeted at pediatric and adolescent populations. In addition, we examined and categorized the methodological issues reported in the reviewed economic evaluations with the purpose of informing methodological areas where future research might need to be prioritized.

## Methods

### Data Sources and Search Strategy

We used free-text terms to search for articles indexed in PubMed (MEDLINE database) from November 2011 to November 2021. This period was selected because of the emerging nature of DHIs, particularly mobile health and eHealth, which have experienced a growth in uptake in recent years. Search term combinations paired “cost-effect*,” “cost benefit,” “cost utility,” “economic evaluation,” “health economic analysis,” “value for money,” “decision model*,” and “cost consequence” with each of “child*,” “paediatric” and “adolescen*,” “infant*,” “neonat*,” “newborn*,” “baby,” and “babies” and each of the following terms: “telemedicine,” “remote* deliver*,” “telehealth,” “digital health,” “mobile health,” “m-health,” “ehealth,” “internet,” and “online” (Textbox 1). The selection of search terms was made based on a review of previously published literature on economic evaluations of DHIs, as well as in the pediatric and adolescent populations [9,10,13,14]. We also manually searched the bibliographies of eligible articles to identify other articles of interest. We conducted a systematic search of articles using the PRISMA (Preferred Reporting Items for Systematic Reviews and Meta-Analyses) checklist and reporting recommendations (Textbox 1 and Multimedia Appendix 1) [15].

**Search strategies.**

**PubMed or MEDLINE**

- Cost effective AND child AND telemedicine
- Cost effective AND child AND remote monitoring
- Cost effective AND child AND remote
- Cost effective AND child AND telehealth
- Cost effective AND child AND digital health
- Cost effective AND child AND digital
- Cost effective AND child AND mobile health
- Cost effective AND child AND mhealth
- Cost effective AND child AND ehealth
- Cost effective AND child AND internet
- Cost effective AND child AND online
- Subsequent iterations substituted “cost effective” for other economic evaluation terms (“cost benefit,” “cost utility,” “economic,” “value,” and “cost consequence”) and substituted “child” for “paediatric” and “adolescent” to yield a total of 198 search terms.

### Inclusion and Exclusion Criteria and Study Selection

**Figure 1:**
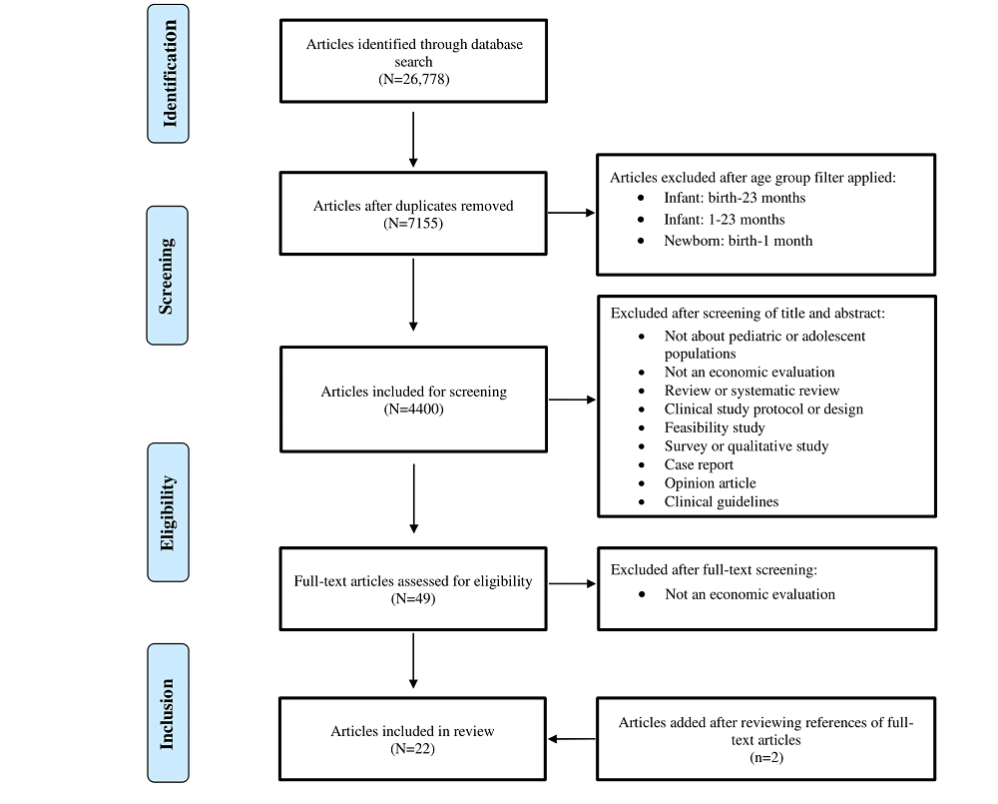
PRISMA (Preferred Reporting Items for Systematic Reviews and Meta-Analyses) study flow diagram. The PubMed search resulted in 26,778 articles from November 2011 to November 2021. Following the removal of duplicates, screening of titles and abstracts and full-text screening based on predefined inclusion and exclusion criteria, a total of 22 articles were selected for inclusion.

In total, 2 reviewers (TS and TSA) independently screened the titles and abstracts of selected articles according to the prespecified inclusion and exclusion criteria: age range of the population of children and adolescents between 1 and 18 years, type of economic evaluation, intervention (ie, telephone, audiovisual consultation, SMS text messaging, mobile phone app, or web-based portal), and outcomes (effects, costs, and cost-effectiveness results). After screening titles and abstracts, we excluded the following types of studies: studies not covering pediatric and adolescent populations, studies that were not economic evaluations, reviews or systematic reviews, clinical study protocols, feasibility studies, surveys, qualitative studies, case reports, opinion articles, and clinical guidelines (Figure 1 and Table 1). In line with the economic evaluation definition commonly used in the literature [16], cost minimization, cost-consequence, and simple cost comparison studies were excluded from this review (Figure 1 and Table 1). We included articles for data extraction if consensus among the reviewers was reached after reviewing the full texts. A third reviewer was used to help resolve any disagreements. The types of economic evaluations that we included in this review were cost-effectiveness analyses (costs per clinical effect), cost-benefit analyses (costs and effects expressed in monetary terms as net benefit), and CUAs (cost per quality-adjusted life year [QALY] or disability-adjusted life year [DALY]) [16,17]. Although cost-per-QALY analyses are a very common type of economic evaluation for adult interventions, they are often not feasible for children and adolescents, primarily because of the methodological challenges and lack of data on valuing health states and deriving QALYs specific to these populations [18,19].

**Table 1:** Inclusion and exclusion criteria.

Category	Inclusion criteria	Exclusion criteria
Language	English	All other languages
Year of publication in PubMed (MEDLINE database)	November 2011 to November 2021	October 2011 and earlier December 2021 and later
Age	1 to 18 years	<1 and >18 years
Article type	Economic evaluation	Studies not covering pediatric and adolescent populations Studies that were not economic evaluations Reviews or systematic reviews Clinical study protocols Feasibility studies Surveys and qualitative studies Case reports Opinion articles Clinical guidelines
Type of economic evaluation	Cost-effectiveness analysis Cost-benefit analysis Cost-utility analysis	Cost minimization Cost-consequence Simple cost comparison

### Data Extraction

We generated a data abstraction procedure (Textbox 2) based on recommendations from the Panel on Cost-Effectiveness in Health and Medicine [20]. A narrative descriptive analysis was used to synthesize the quantitative data. We did not attempt to meta-analyze the results because of the high levels of heterogeneity across the studies. The extracted data of interest included (1) characteristics of the reviewed studies (eg, population, country, disease area, sample size, and DHI type); (2) main results of the studies (costs, outcomes, and incremental cost-effectiveness ratio [ICER]); (3) methodological issues regarding the health economic evaluations; and (4) key cost-effectiveness drivers, which were defined as input parameters that had the most impact on cost-effectiveness results.

To facilitate the summary of the main results of the economic evaluations and comparisons between studies, we converted costs and cost-effectiveness estimates from Great British pound, Euro, and Australian dollar to 2021 US dollar using the following conversion rates: £1=US $1.364, €1=US $1.183, and Aus $1=US $0.751 [21-23].

**Standard operating procedure for data abstraction.**

**Framework and background information**

- Reference (article number, first author, year, title, and journal)
- Diseases under assessment
- Study design (eg, randomized trial or retrospective analysis)
- Perspective from which costs were evaluated (eg, society, patient, health care sector, or a combination of these)
- Interventions
- Comparators
- Tool used (eg, decision tree or analysis, mathematical model, computer simulation model, or expert consultation)

**Data and methods**

- Cost-benefit: what was monetized (eg, lost productivity) and how it was monetized
- Cost-effectiveness: outcomes of interest
- Cost-utility: factors included in utility quantification and how they were quantified in terms of utility
- Description of sensitivity analyses conducted
- Estimates and estimate development

**Inputs**

- Population
  Demographic, behavioral, and clinical characteristics
  Subpopulations, if applicable
  Size
- Location of study (country and setting)
- Costs
  Currency
  Year
  Inflation adjustment method
  Discount rates

**Results**

- Costs and effectiveness results in aggregate
- Costs and effectiveness results disaggregated by groups, if relevant
- Incremental cost-effectiveness ratio
- Variables to which determination of cost-effectiveness was sensitive

**Discussion**

- Distributive effects (ie, who pays for the intervention)
- Recommendations to specific audiences, if any
- Limitations

**References**

- First author, year, and title of relevant articles gathered from references

### Methodological Issues

Most economic evaluations of DHIs follow methodology guidelines used for standard health technologies because of a lack of DHI-specific guidelines and published research [2]. However, DHIs are complex interventions with their own features that pose different types of methodological challenges to economic evaluations compared with pharmaceuticals and medical devices [2]. We reported key methodological challenges and issues concerning the conduct of the reviewed economic evaluations. Drawing on previously published literature [2], we grouped all identified issues into 4 distinct categories related to DHIs, data (clinical or and cost), economic evaluation, and study design.

## Results

### Article Selection

The database search yielded a total of 26,778 articles as of November 2021 (Figure 1). Following the removal of duplicates and studies that did not include populations aged 1 to 18 years, 16.43% (4400/26,778) of the articles remained. We next screened the titles and abstracts and excluded 98.89% (4351/4400) as they were not about pediatric or adolescent populations; were not an economic evaluation; or were a systematic review, clinical study protocol and design, feasibility study, survey and qualitative study, case report, opinion article, or clinical guidelines. We reviewed the full texts of the remaining 49 articles and excluded 29 (59%) as they were not economic evaluations (ie, exclusion of studies that conducted only a simple cost comparison, cost minimization, or cost-consequence analysis). We added 2 articles after screening the references of the reviewed full-text articles. Therefore, we selected 22 articles for inclusion in this review [24-45].

### Overview of Key Characteristics

DHIs administered through an web portal were the most frequent among the included studies (8/22, 36%), followed by SMS text messaging (6/22, 27%) and mobile phone apps (3/22, 14%). Other types of DHIs included telephone consultations (2/22, 9%), audiovisual consultations (2/22, 9%), and web-based symptom monitoring (1/22, 5%). Most studies were conducted in Europe (8/22, 36%) and Africa (6/22, 27%). The most frequently evaluated health care specialties were mental health (7/22, 32%) and maternal, newborn, and child health (MNCH; 7/22, 32%). Other disease areas included asthma, malaria, gastrointestinal disorders, sleeping disorders, child immunization, and conditions requiring emergency care.

Of the reviewed articles, 64% (14/22) included economic evaluations that were based on individual patient data (IPD) [23,24,28,30,32,34-39,41-43], with the remaining focusing on decision analytical modeling (7/22, 32%). Only 5% (1/22) of the economic evaluations used a combination of IPD and decision modeling [31]. Of the 14 IPD-based studies, 13 (93%) were randomized controlled trials, and only 1 (7%) was a non-RCT. The average follow-up duration of the IPD-based and model-based economic evaluations was 11 (range 0.5-36) months and 7 (range 3-10) years, respectively. All studies entailed a cost-effectiveness analysis (6/22, 27%), a CUA (9/22, 41%), or a combination of both (7/22, 32%). In Table 2, we provide a detailed overview of the key characteristics of the studies included in this review.

**Table 2:** Overview of the key characteristics of the economic evaluation studies included in this review.

Article author, year, and journal	DHI^a^ type	Target population	Health care setting	Country	Sample size	Economic evaluation (model or trial based)	Follow-up period (trials) or time horizon (models)
Yang et al [24], 2015, *Medical Decision Making*	Audiovisual consultation	Children presenting to highest triage emergency category	Rural emergency care/pediatric critical care	United States	135	Model based; CEA^b^	5 years
Chatterton et al [25], 2019, *Australian and New Zealand Journal of Psychiatry*	Telephone consultation	Children aged 7-17 years with a diagnosed anxiety disorder	Specialist referral centers	Australia and New Zealand	281	Trial based; CEA and CUA^c^	1 year
Olthuis et al [26], 2018, *Journal of Abnormal Child Psychology*	Audiovisual and telephone consultation	Primary caregivers of children aged 6 to 12 years with disruptive behavior disorders	Community children’s mental health clinics	Canada	172	Trial based; CEA	22 months
LeFevre et al [27], 2018, *JMIR mHealth* *and* *uHealth*	SMS text messaging	Pregnant women and infants	Maternal and neonatal care	South Africa	356	Model based; CUA	5 years
Jo et al [28], 2021, *BMJ Open*	SMS text messaging	Pregnant women	Maternal and neonatal care	Bangladesh	1 million	Model based; CUA	10 years
Willcox et al [29], 2019, *JMIR*	SMS text messaging	Pregnant women, postpartum women, and their children aged <5 years	MNCH^d^ facilities	Ghana	1000	Model based; CUA	10 years
Kawakatsu et al [30], 2020, *Vaccine*	SMS text messaging	Pregnant women as well as children and their parents	Primary care center	Nigeria	9368	Trial based; CEA	3 months
Jo et al [31], 2019, *PLOS ONE*	SMS text messaging	Pregnant women	MNCH facilities	Bangladesh	610	Model based; CUA	4 years
Zurovac et al [32], 2012, *PLOS ONE*	SMS text messaging	Health workers in charge of patients aged <5 years	Pediatric outpatient clinic	Kenya	119	Trial based; CEA	6 months
Modi et al [33], 2020, *JMIR mHealth* *and* *uHealth*	Mobile phone app	Pregnant women and infants	MNCH facilities	India	5754	Model and trial based; CUA	3 years
Bowser et al [34], 2018, *Annals of Global Health*	Mobile phone app	Pregnant women and neonates	MNCH facilities	Nigeria	339,475	Trial based; CUA	1 year
Prinja et al [35], 2018, *Cost Eff and Resource Alloc*	Mobile phone app	Pregnant women and neonates	MNCH facilities	India	300,000	Model based; CUA	10 years
Jolstedt et al [36], 2018, *Lancet Child Adolesc Health*	Web portal	Children aged 8-12 years with a principal anxiety disorder diagnosis	Pediatric mental health centers	Sweden	131	Trial based; CEA and CUA	3 months
De Bruin et al [37], 2016, *Sleep*	Web portal	Adolescents with insomnia	Mental health specialist centers	Netherlands	62	Trial based; CUA	1 year
Lalouni et al [38], 2019, *Clinical Gastroenterol & Hepatology*	Web portal	Children aged 8-12 years with functional abdominal pain disorder	Mental health specialist centers	Sweden	90	Trial based; CEA and CUA	1.5 years
Lenhard et al [39], 2017, *BMJ Open*	Web portal	Adolescents aged 12-17 years with OCD^e^	Mental health specialist centers	Sweden	67	Trial based; CEA and CUA	3 months
Nordh et al [40], 2021, *JAMA Psychiatry*	Web portal	Children and adolescents aged 10 to 17 years with diagnosis of SAD^f^ and their parents	Mental health specialist centers	Sweden	103	Trial based; CEA and CUA	3 months
Aspvall et al [41], 2021, *JAMA Network Open*	Web portal	Children and adolescents aged 8 to 17 years with OCD	Mental health specialist centers	Sweden	152	Trial based; CEA and CUA	10-month trial+6-month follow-up
Lee et al [42], 2017, *Epidemiology and Psychiatric Sciences*	Web portal	Schoolchildren and adolescents aged 11-17 years (subcohort: students with subthreshold depression)	Primary and secondary schools	Australia	23 per class	Model based; CUA	10 years
Sampaio et al [43], 2019, *BMJ Open*	Web portal	Adolescents aged 13-17 years diagnosed with IBS^g^	Primary, secondary, and tertiary care clinics	Sweden	101	Trial based; CEA and CUA	10 weeks
Wasil et al [44], 2021, *J of Consulting & Clin Psychology*	Web portal	Kenyan high school students (regardless of baseline depression symptoms)	High school	Kenya	101	Trial based; CEA	2 weeks
van den Wijngaart et al [45], 2017, *Euro. Respiratory Journal*	Web-based symptom monitoring	Teenagers (aged 12-16 years) and young children (aged 6-12 years) with asthma and their caregivers	Asthma clinic	Netherlands	210	Trial based; CEA	16 months

^a^DHI: digital health intervention.

^b^CEA: cost-effectiveness analysis.

^c^CUA: cost-utility analysis.

^d^MNCH: maternal, newborn, and child health.

^e^OCD: obsessive-compulsive disorder.

^f^SAD: social anxiety disorder.

^g^IBS: irritable bowel syndrome.

### Clinical Effectiveness Estimates

We report key effectiveness outcomes of DHI interventions and comparators in the reviewed studies in Table 3. The most frequently reported health outcomes included the number of deaths averted or lives saved, QALYs, and DALYs. Although most studies (17/22, 77%) suggested that the DHIs were effective, 23% (5/22) of the studies reported not statistically significant differences in health outcomes between the DHI and control groups [25,37,40,41,43]. The most common comparators in the reviewed studies were paper-based, in-person consultations in outpatient settings and the absence of an intervention. Relevant clinical outcomes included care coverage, disease remission rates, immunization rates, treatment response, anxiety and depressive symptoms, and other disease-specific quality-of-life (QoL) metrics. The effectiveness of the DHIs did not seem to differ according to the children’s age cohort (eg, children vs adolescents). Only 5% (1/22) of the studies, which evaluated a web-based asthma monitoring program, reported statistically significant improvements in health outcomes for young children but not for teenagers [45].

**Table 3:** Clinical and economic outcomes of the economic evaluation studies included in this review.

Study	Intervention	Comparator	Effects	Costs (original year and currency)	Costs (converted to 2021 US $)	ICER^a^; DHI^b^ CE^c^ (yes or no); values inside parentheses are converted to 2021 US Dollars to facilitate comparison across studies	Key cost-effectiveness drivers
Yang et al [24]	Telemedicine consultation with remote physician or nurse	Telephone consultation	31% reduction in patient transfers with DHI	Annual cost savings with DHI: US $4662 per patient (2013)	Annual cost savings with DHI: US $5423 per patient	Cost savings: US $46,620 (US $54,227) per ED^d^ per 10 pediatric patients; CE: yes (dominant from payer perspective)	Reduction in ambulance patient transfer rates
Chatterton et al [25]	Stepped care: telephone-delivered 3-stage CBT^e^	Empirically validated face-to-face CBT program with a therapist	No statistically significant QALY^f^ differences between the 2 study arms	Mean cost savings through stepped care: Aus $1334 in total societal cost, Aus $198 in intervention delivery, and Aus $563 in health sector costs (2015)	Mean cost savings through stepped care: US $1249 in total societal cost, US $185 in intervention delivery, and US $527 in health sector cost	Reported visually as a CE plane; CE: yes (dominant from social perspective)	Reduction in indirect (caregiver) costs and increase in insurance reimbursement availability (Medicare)
Olthuis et al [26]	Written material, skill-based videos, and telephone coaching sessions for caregivers	Usual care—mental health services offered by referring agency or other providers	0.56 improvement with DHI in child behavior checklist scores	Cost savings with DHI: CAD $1059 (2016)	Cost savings with DHI: US $861	Average bootstrapped ICER: −US $2128 (US $1730) of DHI compared with usual care; CE: yes (dominant)	Reduction in costs of educational and health care services
LeFevre et al [27]	SMS text messaging service for pregnant women	No intervention	95% vs 90% immunization rate in DHI vs control group	US $1.2 million 5-year DHI cost (2015)	US $1.37 million 5-year DHI cost	US $1985 (US $2269) per DALY^g^ in first year; US $200 (US $229) per DALY in fifth year; CE: yes	Increase in number of lives saved and reduction in programmatic costs
Jo et al [28]	Comprehensive and basic pregnancy surveillance intervention	SOC^h^; paper based	3076 averted deaths in 10 years with DHI	US $43 million (US $115 million) incremental (societal) DHI cost (2018)	US $46.4 million incremental DHI cost and US $124 million incremental societal DHI cost	US $327 (US $353) and US $462 (US $499) per DALY averted; CE: yes	Increase in number of lives saved, population coverage, and implementation duration and reduction in program costs
Willcox et al [29]	Interactive voice messages on pregnancy and infant care; appointment reminders for clinical visits	SOC	59,906 lives saved and cumulative 1,550,028 DALYs averted in 10 years with DHI	DHI cost: US $66,166 per district per year (2014)	DHI cost: US $75,734 per district per year	US $20.94 (US $23.97) per DALY averted and US $586.72 (US $671.56) per death averted; CE: yes	Reduction in still deaths and maternal deaths, personnel time, and program start-up costs (training and equipment)
Kawakatsu et al [30]	SMS text message reminder 2 days before in-person appointments	No intervention	4.8%-6% increase in return rate with DHI	DHI development: US $26,466 (65%); mobile phones: US $6314 (2019)	DHI development: US $28,051 (65%); mobile phones: US $6629	US $7.90 (US $8.40) per return case; CE: N/A^i^	Reduction in number of appointments and increase in geographic coverage of SMS text message reminders
Jo et al [31]	Comprehensive pregnancy surveillance intervention	Basic pregnancy surveillance	354 averted newborn deaths per 1 million with DHI	Total 2-year incremental cost of DHI: US $319,000 (2016)	Total 2-year incremental cost of DHI: US $360,154	US $31 (US $35) per DALY averted and US $901 (US $1017) per death averted; CE: yes	Reduction in program costs (mainly supervision and training); increase in population coverage
Zurovac et al [32]	SMS text message reminders sent to health workers on pediatric malaria case management	No intervention	25% of additional children correctly managed; additional number of febrile children correctly managed—under study conditions: 38,435, under implementation by the Ministry of Health: 38,435, and under national implementation: 2,955,250	Total costs—under study conditions: US $19,342, under implementation by the Ministry of Health: US $13,920, and under national implementation: US $97,350 (2010)	Total costs—under study conditions: US $24,036, under implementation by the Ministry of Health: US $17,298, and under national implementation: US $120,973	Cost per additional child correctly treated—under study conditions: US $0.50 (US $0.62), under implementation by the Ministry of Health: US $0.36 (US $0.45), and under national implementation: US $0.03 (US $0.04); CE: yes	Results robust to changes in input parameters in sensitivity analyses
Modi et al [33]	Mobile phone app reminders; health promotion and decision support with web interface	SOC	11 averted infant deaths per 1000 live births with DHI	Annual incremental cost of DHI: US $163,841 (2016)	Annual incremental cost of DHI: US $184,978	US $84 (US $95) per life years saved and US $5709 (US $6446) per death averted; CE: yes	Increase in district scale-up and program effectiveness
Bowser et al [34]	Mobile devices, phones, and tablets for case management and decision support	No intervention	Higher care coverage and 4661 lives saved with DHI, including women, neonates, and stillbirths	Incremental cost savings with DHI: US $610 (2014)	Incremental cost savings with DHI: US $699	US $13,155 (US $15,057) per life saved and US $568 (US $650) per DALY averted; CE: no (in base case)	Number of unassisted deliveries
Prinja et al [35]	Routine care+mobile phone app used by community health workers in MNCH^j^ care	SOC	Reduction of 0.2% and 5.3% in maternal and neonatal deaths, respectively, over 10 years with DHI	Incremental cost of DHI: US $982 million (2015)	Incremental cost of DHI: US $1.1 billion (of which 90% implementation)	Health system perspective: US $205 (US $234) per DALY averted and US $5865 (US $6705) per death averted; societal perspective: DHI is cost saving; CE: yes	Increase in uptake of preventive services and reduction in number of maternal and neonatal illnesses
Jolstedt et al [36]	iCBT^k^	Internet-delivered child-directed play	48% vs 15% remission rate in DHI vs control	Average societal cost saving with DHI: €493 (2016)	Average societal cost saving with DHI: US $606	ICER not calculated because of minimal differences in QALYs (0.02 years); CE: yes for CEA^l^ and no for CUA^m^	Reduction in intervention costs
De Bruin et al [37]	iCBTI^n^	fCBTI^o^	No significant differences in sleep efficiency and quality of life	No significant cost differences	No significant cost differences	DHI intervention dominates; CE: yes	Reduction in intervention costs and ongoing intervention costs (after trial period) and reduction in willingness-to-pay threshold
Lalouni et al [38]	iCBT	Usual treatment (in health and school system)	Significant and substantial improvement in gastrointestinal symptoms, quality of life, and avoidance behaviors in DHI group	Average societal cost savings per patient with DHI: US $974 (2016)	No significant cost differences	DHI intervention dominant; US $1050 (US $1186) cost savings per patient treated with DHI; CE: yes	Results robust to changes in input parameters in sensitivity analyses
Lenhard et al [39]	iCBT	Untreated condition (patients on waitlist)	27% and 0% treatment response in iCBT and control group, respectively	Average societal cost savings per patient with DHI: US $145 (2016)	Average societal cost savings per patient with DHI: US $164	Societal perspective: iCBT dominant; health care perspective: ICER of US $78 (US $86) per responder; CE: yes	Reduction in health care resource use
Nordh et al [40]	iCBT	iSUPPORT^p^—active comparator	Nonsignificant QALY differences	Average societal cost savings per patient with DHI: €1076 (2018)	Average societal cost savings per patient with DHI: US $1393	ICER: €17,901 (US $23,167) per QALY (iCBT dominant over the active comparator); CE: yes; however, from HCP^q^ perspective, iCBT more costly but more effective	Reduction in education costs and increase in school productivity
Aspvall et al [41]	Guided iCBT implemented within a stepped-care model (iCBT+in person)	In-person CBT	68% treatment response in both groups; mean QALY difference=−0.029	Average cost savings per patient with DHI: US $2052 (2020)	Average cost savings per patient with DHI: US $2148	Mean cost savings of US $2104 (US $2203) per participant (39% relative savings) from health care sector perspective and US $1748 (US $1830) per participant from societal perspective; CE: yes	Not reported
Lee et al [42]	(1) Internet-delivered depression prevention—uDHI^r^ and iDHI^s^ and (2) face-to-face depression prevention—uF2F^t^ and iF2F^u^	No intervention	uF2F: 3367 DALYs averted; iF2F: 4083 DALYs averted	Incremental net cost of DHI: Aus $37,041 (2013)	Incremental net cost of DHI: US $44,719	uF2F: ICER of Aus $7350 (US $8874) per DALY averted; iF2F: ICER of Aus $19,550 (US $23,602) per DALY averted; uDHI and iDHI were highly cost-effective when assuming 50%-100% relative effect size compared with F2F; CE: yes	Increase in intervention effect size and long-term health impacts and reduction in intervention costs and indirect costs (time and travel)
Sampaio et al [43]	Exposure-based iCBT	Waitlist control	iCBT group had small QALY gains (0.0031) and average improvement of 5.647 points on PedsQL^v^ compared with control	Average incremental cost of DHI per participant: US $170 (2016)	Average incremental cost of DHI per participant: US $192	CUA: ICER of US $54,916 (US $62,001) per QALY gained with DHI; CEA: US $85.29 (US $96.29) per PedsQL point improvement with DHI; CE: undetermined	Reduction in intervention costs and resource use
Wasil et al [44]	Online single-session depression intervention	Online study skills active control	Greater reduction in depressive symptoms in DHI group (PHQ-8^w^ score standardized mean difference=0.5)	Incremental cost of DHI per student: US $3.6 (2020)	Incremental cost of DHI per student: US $3.77	US $25.35-$34.62 (US $26.54-$36.25) per case; CE: N/A	Reduction in cost components
van den Wijngaart et al [45]	VAC^x^—outpatient visits reduced by 50% and monthly web-based asthma control test for monitoring	Usual care—routine 4-monthly outpatient visits including an ACT^y^	Asthma control higher with VAC than usual care for young children (mean difference=1.17); nonsignificant difference for teenagers	Mean cost saving per patient: €352 for young children and €852 for teenagers (2014)	Mean cost saving per patient: US $556 for young children and US $1345 for teenagers	DHI dominant in all subcohorts for asthma control outcome and in caregiver subcohort for quality-of-life outcome; CE: yes	Number of outpatient clinic visits and reduction in travel expenses

^a^ICER: incremental cost-effectiveness ratio.

^b^DHI: digital health intervention.

^c^CE: cost-effective.

^d^ED: emergency department.

^e^CBT: cognitive behavioral therapy.

^f^QALY: quality-adjusted life year.

^g^DALY: disability-adjusted life year.

^h^SOC: standard of care.

^i^N/A: not applicable.

^j^MNCH: maternal, neonatal, and child health.

^k^iCBT: internet-delivered CBT.

^l^CEA: cost-effectiveness analysis.

^m^CUA: cost-utility analysis.

^n^iCBTI: iCBT for insomnia.

^o^fCBTI: face-to-face CBT for insomnia.

^p^iSUPPORT: internet-based supportive therapy.

^q^HCP: health care professional.

^r^uDHI: universal DHI.

^s^iDHI: indicated DHI.

^t^uF2F: universal face-to-face depression prevention.

^u^iF2F: indicated face-to-face depression prevention.

^v^PedsQL: Pediatric Quality of Life Inventory.

^w^PHQ-8: 8-item Patient Health Questionnaire.

^x^VAC: virtual asthma clinic.

^y^ACT: asthma control test.

### Cost Estimates

We summarized the cost estimates associated with DHI implementation and the associated health care costs for children and adolescents. Further details regarding the exact cost components included in each of the reviewed studies can be found in Table 4. Many (9/22, 41%) economic evaluation studies took both health system and societal perspectives, whereas the remaining studies were distributed evenly between the health care system and societal perspectives.

**Table 4:** Estimates of direct and indirect costs associated with the digital health interventions (DHIs) included in this review.

Study	DHI type	Currency and year	Study perspective	Cost components	Non–health care costs included?
Yang et al [24]	Audiovisual consultation	US dollar, 2013	Health care payer	Telemedicine operational cost per child or emergency department Emergency department visit cost Patient transfer cost: air or ground ambulance Hospital treatment cost: rural or community or tertiary	No
Chatterton et al [25]	Telephone consultation	Australian dollar, 2015-2016	Health care system and societal	DHI intervention (standard, advanced, and phone therapist and self-help print and audio material) Medical service (GP^a^, pediatrician, practice and mental health nurse, psychiatrist, psychologist, social worker, community health services, and school counselor) Medications Indirect costs (productivity losses from DHI intervention captured through parental time off from work and leisure hours)	Yes
Olthuis et al [26]	Audiovisual and telephone consultation	Canadian dollar, 2016	Health care system	DHI intervention: employee salaries (management, evaluators, coaches, administration, and programmers) and operational costs (rent, utilities, intervention materials, office supplies, telecommunications, postage, licenses, training, and legal and insurance costs) Medications: self-reported medications by families (brand or lowest-cost generics) Health care services (compensation to health care service providers through collective bargaining agreements and overnight stays in mental health facilities) Educational services (compensation to educational service providers)	No
LeFevre et al [27]	SMS text messaging	US dollar, 2015	Societal	Implementation (including development, start-up, training, personnel, buildings, transport, and communication) Technology (content and technology maintenance, monitoring, project management, SMS text messaging delivery, travel, and printing) Indirect costs (wages lost resulting from time spent seeking care)	No
Jo et al [28]	SMS text messaging	US dollar, 2018	Societal	Program cost—digital system: partnerships and census building, system optimization, phone and tablet procurement, survey printing, supervision, pregnancy surveillance, SMS text message reminders, census enumeration, data reporting and processing, and server hosting Program cost—paper system: including survey or registry printing, training, supervision, census enumeration, pregnancy surveillance, and data reporting and processing Provider and user unit costs (including antenatal care, home delivery, facility delivery, and postnatal care)	No
Willcox et al [29]	SMS text messaging	US dollar, 2014	Health care payer or program	Development (including program design, telecommunications, technology, and personnel) Start-up (including district profiling, content localization, equipment, customer support, training, community mobilization, partnership building, vehicle and office maintenance, telecommunications, technology, and personnel and benefits) Implementation (including technical groups, monitoring and evaluation, continued training, equipment and materials, vehicle and field office maintenance, telecommunications, technology maintenance, and personnel and benefits)	No
Kawakatsu et al [30]	SMS text messaging	US dollar, 2019	Health care payer or government	Start-up costs (development of the API^b^ portal, administrative web portal, and mobile app) Recurrent or operation costs (fee for maintenance and adjustment of server and app, SMS text message reminders, default tracing services, and data communication)	No
Jo et al [31]	SMS text messaging	US dollar, 2016	Program	Program development (mobile phone procurement and system development) Start-up (training and community outreach) Implementation (supervision, mobile phone–based pregnancy surveillance, server maintenance, SMS text message reminders, and visit reminders)	No
Zurovac et al [32]	SMS text messaging	US dollar, 2010	Program implementer	Intervention development and refinement (employee salaries and subsistence for senior researcher, research assistant, pretesting and reviews of SMS text messages, and mileage cost of transport) Distribution system development (consultation fee, project computer, modems, purchase of postpaid phone numbers, and airtime for testing distribution systems) Cost of collecting health workers’ phone numbers (vehicle, research assistant, driver, district public health nurse, and traveling subsistence) Implementation (total cost of sending SMS text messages and research assistant)	No
Modi et al [33]	Mobile phone app	US dollar, 2016-2017	Health care payer (government)	Start-up cost (including software development, vehicles, mobile handset, other IT equipment, and training) Implementation cost (including personnel, training, software annual development and maintenance, travel, and IT and office expenses)	No
Bowser et al [34]	Mobile phone app	US dollar, 2014	Societal	Capital (eg, buildings, equipment, and vehicles) Recurrent (nonmedical consumables [eg, water and electricity] and medical consumables [eg, drugs, test kits, and vaccinations]) Personnel (salaries of clinical and nonclinical staff) Societal (direct nonmedical costs included patients’ out-of-pocket expenditures on transportation and food associated with obtaining an antenatal visit at a health center; indirect costs were the monetary value of days of school lost and income lost because of the antenatal visit incurred by patients and their caregivers)	Yes
Prinja et al [35]	Mobile phone app	Indian rupee, 2015	Health care system and societal	Start-up (software development, training of community health workers, equipment, purchase of mobile phones, programmatic expenses, overheads, and administrative) Implementation (M&E^c^, preventive services, and curative services) Health system (unit costs for antenatal, postpartum, neonatal, and pediatric care)	Yes
Jolstedt et al [36]	Online portal	Euro, 2016 (converted from Swedish krona)	Societal	Health care consumption (physician and psychologist appointments) Supportive resources (eg, study help, medication, and dietary supplements) Intervention (individual per-participant therapist times and online platform maintenance costs, ie, IT support, server costs, and software updates) Societal (child’s absence from school, parents’ absence from work, and productivity losses)	Yes
De Bruin et al [37]	Online portal	Euro, 2014	Societal	Resource use—user/family perspective (physician visits, use of medication, mental health care visits, and additional help at school/home) Direct and indirect non–health care costs—user/family perspective (informal care, parents’ loss of (non)paid work, traveling expenses, and tutoring of the adolescent) Program costs—internet and group CBTI^d^ (therapists’ registered hours spent preparing and delivering consults, for administrative purposes, and for intervision and supervision sessions)	Yes
Lalouni et al [38]	Online portal	US dollar, 2016	Societal	Health and health care use (GP, nurse, counselor, psychotherapist, psychologist, specialist practitioner, dietician, and dentist) Medications (pain, laxatives, gastrointestinal, and probiotics) Intervention (online platform maintenance and iCBT therapist wage) Productivity losses (missed days at school for children and at work for caregivers and foregone leisure)	Yes
Lenhard et al [39]	Online portal	Swedish krona, 2014, and US dollar, 2016	Societal and health care payer	iCBT^e^ intervention (clinicians’ time for the 12 weeks of iCBT and iCBT treatment platform maintenance) Health care resource use and supportive resources (medical doctor or psychologist visits and private tutoring) Medications (prescriptions, prescription-free drugs, or supplements) Societal costs (absenteeism from school and academic and parental productivity loss)	Yes
Nordh et al [40]	Online portal	Swedish krona and Euro, 2018-2019	Societal, health care payer, and health care professional	Health and health care use (GP, nurse, counselor, psychotherapist, psychologist, specialist practitioner, dietician, and dentist) Medications (pain, laxatives, gastrointestinal, and probiotics) Intervention (online platform maintenance and iCBT therapist wage) Productivity losses (missed days at school for children and at work for caregivers and foregone leisure)	Yes
Aspvall et al [41]	Online portal	US dollar, 2020	Societal and health care sector	iCBT intervention (clinicians’ time/salary) Health care resources (GP, nurse, counselor, specialist practitioner, psychotherapist, psychologist, speech and language therapist, and dietician) Supportive resources (specialist teacher, personal assistant, and study help) Medications (prescriptions, prescription-free drugs, or supplements) Societal costs (absenteeism from school, academic and parental productivity loss, and support from family and friends)	Yes
Lee et al [42]	Online portal	Australian dollar, 2013	Health care payer (public sector) and societal	Health system (screening of depression symptoms and further testing and annual cost of a prevalent depression case) Interventions (salaried staff and psychologist wages, training of teachers to deliver face-to-face interventions, annual subscription to online portal, and teacher supervision for internet-delivered interventions) Cost offsets (depression treatment costs averted through prevention) Productivity costs (decreased labor productivity or absenteeism attributable to poor health; in sensitivity analysis, travel costs for patients)	Yes
Sampaio et al [43]	Online portal	Swedish krona, 2016, converted to US dollar, 2016	Societal and health care system	Intervention (salaried time for therapists to give support to families and online platform maintenance cost) Medications, costed using market prices (pharmaceuticals and prescription-free medications, ie, dietary supplements) Health care resource use, costed using Swedish national tariffs (specialist practitioner, psychologist, and medical technology staff) Societal (indirect) costs (productivity losses because of efficiency loss and absenteeism from school [adolescents] and from paid and unpaid work [parents] and foregone leisure time)	Yes
Wasil et al [44]	Online portal	US dollar, 2020	School, researcher, and societal	School costs (teachers’ time required to oversee the administration of the intervention and provision of internet) Intervention/researcher costs (hosting the DHI on a website and backup internet data) Societal costs (school+intervention)	Yes
van den Wijngaart et al [45]	Online symptom monitoring	Euro, 2014	Societal and health care system	Health care/direct costs associated with medical conditions (GP consultations, pediatrician consultations, other specialists, physiotherapists, consultations by phone, emergency room visits, and hospital admissions) and prescribed medication Societal/indirect costs (loss of productivity, travel costs for any medical condition, and parking expenses) Intervention (VAC^f^ development and estimated hosting and license costs)	Yes

^a^GP: general practitioner.

^b^API: application programming interface.

^c^M&E: monitoring and evaluation.

^d^CBTI: cognitive behavioral therapy for insomnia.

^e^iCBT: internet-delivered cognitive behavioral therapy.

^f^VAC: virtual asthma clinic.

Most studies (18/22, 82%) reported detailed direct cost components broken up into 3 phases of integrating the intervention into the health care system: DHI development, DHI start-up, and DHI implementation. Approximately 55% (12/22) of the studies reported indirect costs, which typically included productivity losses because of missed hours from school for children and from paid and unpaid work for parents and caregivers, parental out-of-pocket expenditures on travel, informal care, and educational expenses associated with tutoring and mentoring of the target population.

DHIs were associated with cost savings compared with the standard of care in 45% (10/22) of the studies [24-26,34,36,38-41,45], ranging from a mean of US $164 to US $5423 per patient (Table 3) [24,39]. Other studies reported the cumulative annual incremental cost of DHIs compared with the standard of care, which ranged from US $184,978 to US $1.1 billion depending on the scale-up of the intervention program [33,35] (Table 3). The relative proportion of each of the DHI-related cost components (reported only in 9/22, 41% of the studies) was as follows: 12% average start-up cost (range 5%-24%), 15% average development cost (range 6%-23%), and 73% average implementation cost (range 63%-89%) [24,27-31,33-35].

### Cost-Effectiveness Outcomes

We reported key findings of the economic evaluations in Table 3 grouped by DHI type. In 82% (18/22) of the reviewed studies, DHIs were cost-effective (15/18, 83%) or cost saving (3/18, 17%) compared with the standard of care, with an ICER ranging from US $24 to US $23,602 per DALY averted [29,40,42,43]. In 32% (7/22) of the studies, DHIs were a dominant strategy.

### Cost-Effectiveness Drivers

The most frequent drivers of cost-effectiveness across the studies were population coverage, cost components, and intervention effect size (Table 3). In particular, the cost components associated with the implementation of DHI programs and supervision/training of health care professionals had the most impact on cost-effectiveness results [26-29,36,37,40,42-44]. For instance, the cost-effectiveness of internet-delivered universal prevention of major depression was highly sensitive to variations in implementation cost (ie, small changes to the average implementation cost per person led to large changes in the resulting ICER, with the intervention becoming not cost-effective when such cost was >US $90 per person [42]).

The mean differences in health outcomes between DHIs and comparators were most sensitive to factors such as population coverage [28,30,35], assumptions about the number of deaths averted or lives saved [27-29], and number of clinic visits [30,45]. For example, the annual coverage increase from 5% to 10% for antenatal and postnatal care was associated with twice as many lives saved over 10 years of implementation of an SMS text messaging program for pregnant women attending MNCH facilities [28].

### Overview of Methodological Issues

#### DHI-Related Methodological Issues

Challenges directly related to data collection or modeling of the evaluated DHI interventions were reported in 36% (8/22) of the articles and included the following types of issues (Table 5):

1. *Lack of a well-defined comparator* was observed in 75% (6/8) of the studies [26,35,36,38,40,45]. This included potential biases arising from differences in the delivery format of the DHI and comparator interventions [26] as well as the mixing of DHI and comparator interventions in certain child groups [35,45].
2. *Lack of user involvement* was reported to weaken the effectiveness of DHI interventions. This was mostly because of difficulties in maintaining the expected level of user involvement during the study period that would be desirable for the specific DHI [24,25,30].
3. *Difficulty in measuring the impact of DHIs on costs and outcomes.* This was because of either the complexity of DHI programs that was not captured by the economic evaluation [28,31] or the lack of a clear understanding of the pathways to impact, where outcomes may have been influenced by non-DHI components (eg, complementary services) [31].

**Table 5:** Summary of the limitations and methodological issues of the reviewed studies.

DHI^a^ type, reference, and type of methodological issue			Description
**Audiovisual consultation [24]**			
	Study design	Low generalizability; selection bias—telephone and telemedicine consultations not randomly assigned; children in DHI group were younger than those in the control group	
	DHI related	Low telemedicine use and likely overestimated operation cost because of small cohort	
	Clinical data	No patient follow-up data to monitor potential postdischarge health problems	
**Telephone consultation [25]**			
	Study design	Low generalizability (a single specialist referral center with high socioeconomic status); measuring differences in clinical outcomes but not in cost outcomes; double counting of parental time costs	
	Clinical data	Some information collected in self-reported questionnaires was subject to recall bias	
**Audiovisual and telephone consultation [26]**			
	Clinical data	Parent self-reported measures leading to incidental misreporting because of memory errors (long trial period); no data collected on diagnostic remission; missing demographic data for a large percentage of the sample	
	Cost data	Costs associated with accessing mental health services not included	
	DHI related	Inconsistency between treatment arms as DHI was delivered one-to-one and usual care was delivered in group format	
	Study design	No blinding to random allocation; DHI self-selection bias; generalizability limited (narrow age range)	
**SMS text messaging [27]**			
	Clinical and cost data	Lack of primary data (patient recruitment challenges); incomplete data records for approximately 50% of participating women upon exit interviews	
	DHI related	Most of the fixed costs of DHI did not vary with changing program scale	
**SMS text messaging [28]**			
	DHI -related	Limited empirical data and evidence on large-scale mHealth^b^ programs for pregnancy; thus, numerous assumptions about population and service coverage inputs	
	Economic evaluation	Model does not incorporate complexities between preventive and curative care	
**SMS text messaging [29]**			
	Cost data	Cost data collected from a single district and did not include costs incurred by pregnant or postpartum women to seek care or to the health system to collect data	
	Study design	Methodological weaknesses in study design and data collection methods (sampling and survey tool)	
**SMS text messaging [30]**			
	Study design	Number of participants divided into 2 groups was not equal or adequately balanced	
	DHI related	Study not able to verify whether SMS text message reminders were received and further read by clients in the DHI group	
**SMS text messaging [31]**			
	Study design	Quasi-experimental design—not enough statistical power and adjustment for confounding factors	
	Cost data	Cost adjustment for standardized estimations to 1 million population may not incorporate changes with scaling up; household and service provision costs associated with DHI not included	
	DHI related	Intervention was a reminder, not provision of care, implying that the health outcome could have been influenced by other determinants of access and quality of care in the system	
**SMS text messaging [32]**			
	Study design	Short follow-up; new “test and treat” malaria case management policy not tested under trial conditions; patients aged ≥5 years not included	
**Mobile phone app [33]**			
	Cost data	Did not assess health care input cost or time spent by health workers in training and supportive supervision by medical officers and health care professionals; total cost of implementing DHI assumed to be the same in per-protocol and intention-to-treat analyses, which may be an overestimation in the per-protocol analysis	
**Mobile phone app [34]**			
	Cost and clinical data	Not able to track provision of services for all 10 antenatal care interventions	
	Clinical data	No follow-up data to monitor compliance with hypertension management; no appropriate data to track subsequent second tetanus toxoid vaccination	
	Economic evaluation	Assumed full compliance of women given iron folate and malaria prophylaxis	
**Mobile phone app [35]**			
	DHI related	Not clear whether the effect of several simultaneous interventions was additive, multiplicative, or otherwise	
	Cost data	Intervention scale-up costs estimated in ideal conditions without bottlenecks in implementation	
	Study design	Intervention was not randomly assigned, leading to possible confounding	
**Web portal [36]**			
	Clinical data	Available measures of quality of life may lack validity for children and adolescents with anxiety disorders	
	Study design	Active control condition rated as being less credible at week 3; results may not be generalizable to the entire patient population (most patients were self-referred and from educated families); participants with missing data were more severely ill at baseline and in the comparator vs DHI group; short follow-up	
**Web portal [37]**			
	Cost data	Uncertainties on ongoing vs sunk costs; ongoing costs of DHI may have been overestimated because of small sample size	
	Study design	No conclusions on noninferiority can be drawn (this was an intention-to-treat rather than per-protocol analysis)	
**Web portal [38]**			
	Study design	Crossover from usual treatment to iCBT^c^ after 10-week follow-up; high educational level of parents may reduce external validity; inclusion criterion of basic reading and writing skills excluded newly arrived or marginalized immigrants; not possible to blind patients and therapists to treatment assignment	
**Web portal [39]**			
	Study design	Moderate sample size; measurements at 2 time points (before and after the intervention); short follow-up	
**Web portal [40]**			
	Study design	Most participants were self-referred, with potential confounding effects of higher motivation to work compared with typical patients with SAD^d^	
	Economic evaluation	Comparator less credible than iCBT	
**Web portal [41]**			
	Cost data	Tax-funded universal health system in Sweden may affect interpretation of the results; other health care resources and societal costs were assessed retrospectively with a parent-reported measure	
	Study design	Stepped care may result in delayed treatment response and, thus, is not the preferred choice for policy makers	
**Web portal [42]**			
	Study design	Study narrowly focused on health benefits linked to prevention of incidence of depression only	
	Economic evaluation	Model assumed that preventive interventions for depression led to a reduction in depression incidence based on the outcomes of meta-analyzed RCT^e^ studies with short time frames; excluded evidence from RCT studies assessing depression symptom changes	
	Cost and clinical data	Data limitations (old intervention pathways, effectiveness data with high risk of bias, and lack of cost data)	
**Web portal [43]**			
	Economic evaluation	Short time horizon because of lack of evidence on longer-term effects of DHI; no active comparator	
	Clinical data	No multi-attribute utility instrument for dimensions affected by IBS^f^ in adolescents	
**Web portal [44]**			
	Cost data	Cost data estimated retroactively; prospective monitoring of costs could yield more precise estimates	
	Study design	Short follow-up data collection period (2 weeks)	
**Web-based symptom monitoring [45]**			
	Study design	Frequency of outpatient visits may differ in clinical practice compared with RCT conditions	
	DHI related	DHI partly combined with usual care in the intervention arm	

^a^DHI: digital health intervention.

^b^mHealth: mobile health.

^c^iCBT: internet-delivered cognitive behavioral therapy.

^d^SAD: social anxiety disorder.

^e^RCT: randomized controlled trial.

^f^IBS: irritable bowel syndrome.

#### Issues With Measuring Costs and Outcomes

Limitations of measuring outcomes included biases in self-reported patient information, lack of primary data on resource use, and lack of appropriate QoL measures adjusted to younger age groups (ie, use of generic rather than pediatric EQ-5D and 36-item Short Form Health Survey instruments). The most frequent challenges with cost data were incomplete resource use data, costing intervention scale-up because of difficulties in estimating the likely population size, and uncertainties regarding distinguishing and measuring operational costs versus sunk costs.

#### Economic Evaluation Methodological Assumptions

We identified methodological issues related to decision modeling in 23% (5/22) of the economic evaluation studies [28,34,40,42,43]. This included failure to appropriately model preventive and curative care in complex treatment pathways, short model time horizons because of lack of evidence on longer-term effects of DHIs, and issues with the validity of the input parameters (Table 5). Another prevalent issue with modeling of DHIs in child settings was the difficulty of summarizing results into a single metric, such as cost per QALY, because of the lack of generic health outcomes in this population [25,32,41] (Table 2).

#### Study Design Issues

The reviewed studies also faced methodological issues related to the study design (17/22, 77%). These issues primarily related to studies that used individual patient-level data (14/17, 82%) rather than decision analytic models. At the clinical trial initiation/enrollment stage, these issues included selection bias and the absence of blinding to random allocation. For instance, 12% (2/17) of the studies faced potential confounding because of age differences between the intervention and control groups [24,35]. In addition, participants in some of the studies were permitted to self-refer, resulting in self-selection bias [24,26,40]. Other limitations related to the study design included low generalizability/external validity, small sample size because of the difficulty of recruiting children and adolescents for DHI programs, short study follow-up period, and incomplete data because of loss to follow-up (Table 5). Low generalizability was also a common issue in the included studies [24-26,36]. For instance, a narrow age range of the trial population posed a barrier to generalizing the findings of the economic evaluations to a wider health care setting or patient population [26].

## Discussion

### Principal Findings

This systematic review identified 22 economic evaluations of DHI interventions for children and adolescents. Most studies (14/22, 64%) evaluated interventions delivered through online portals or SMS text messaging, most frequently within the health care specialties of mental health and MNCH. In 82% (18/22) of the reviewed studies, the DHI was cost-effective or cost saving compared with the standard of care. The studies reported various levels of granularity of cost components used in the economic evaluations; however, most studies (18/22, 82%) included direct medical costs and DHI costs broken up into 3 major components: DHI development, DHI start-up, and DHI implementation. More than half (12/22, 55%) of the reviewed studies also included indirect costs, such as productivity losses because of missed hours from school for children and from paid and unpaid work for parents and caregivers. The methodological challenges commonly identified in the reviewed studies suggest that issues with study design were the most prevalent (17/22, 77%), followed by issues with cost data (11/22, 50%) and challenges related to decision modeling (5/22, 23%). The most frequent drivers of cost-effectiveness included population coverage, implementation and user support costs, and intervention effect size.

### Comparison With the Existing Literature and Method Guidelines

This study provides the first systematic review of economic evaluations of DHIs targeting pediatric and adolescent populations. In line with the published literature on adult populations, we found that many of the economic evaluations of DHIs for children and adolescents focused on the management of long-term conditions and mental health, primarily in the form of CBT, cardiac monitoring, and weight loss and diabetes management [5,11,46,47]. In these areas, our findings are very much in line with those for adult populations. For example, our review found SMS text message reminders used for weight loss management to be cost-effective, similar to findings for adult populations [11]. However, we identified other areas where DHIs for children and adolescents are often cost-effective or cost saving compared with usual care in contrast to related literature in adult settings [11]. For example, videoconferencing sessions for children and adolescents were found to be cost-effective, whereas similar videoconferencing interventions in adult populations are rarely cost-effective, as well as having no significant impact on health-related QoL [11].

Most of the studies (19/22, 86%) included in this review are unlikely to comprehensively meet the evidence standards framework published by the National Institute for Health and Care Excellence (NICE) and the World Health Organization [3,48]. For example, NICE guidelines suggest the need for generalizability of economic evaluation assumptions beyond the local context, but most of the reviewed studies (15/22, 68%) focused on a narrow patient population with specific characteristics that are not directly generalizable. In addition, many of the reviewed studies (12/22, 55%) did not have a sufficiently long time horizon to capture the relevant health outcomes and costs and did not provide a clear justification for the chosen sensitivity analysis as per NICE recommendations [48].

### Strengths

This review adds to the current body of literature by identifying some key methodological issues in the reviewed economic evaluations that are relevant to the pediatric setting. These include mixing of DHI and comparator interventions across treatment arms, inconsistencies in accounting for different cost components of DHIs (eg, maintenance, sunk, and scale-up costs), and difficulties maintaining user involvement.

The methodological issues specific to the nature of DHIs and the difficulties of comparing economic evaluations of DHIs because of heterogeneity align well with those previously identified in the published literature [1,2,11,49,50]. These include lack of established measures for clinical effectiveness of DHIs, difficulty in distinguishing DHIs from “standard of care,” and inability to measure and model broader costs and effects of DHIs beyond the direct impact on the health system (ie, production losses, travel costs, absenteeism, and presenteeism for parents and caregivers). In the context of the published literature, many of the study design issues were similar to those faced in clinical studies of health care interventions more broadly and included selection bias, small sample size, low generalizability, short or no follow-up period, and no blinding to random allocation.

### Limitations

This systematic review has some limitations. The database search was limited to studies published between 2011 and 2021 and indexed in PubMed. Considering that DHIs have received increasing attention in the last decade, this time restriction may be reasonable. This review was based on the MEDLINE database and has not been comprehensively combined with other databases. However, when we performed a high-level search in Embase, it did not retrieve any additional relevant studies, suggesting that the PubMed search strategy likely covered the vast majority of the relevant economic evaluation studies.

This review did not assess the reporting quality of the reviewed studies in line with reporting guidelines such as CHEERS (Consolidated Health Economic Evaluation Reporting Standards) [51] for 2 reasons: first, published economic evaluations are typically required to follow the CHEERS checklist, and second, our *quality assessment* focused on a wider range of methodological issues regarding economic evaluations, which included aspects more relevant to the conduct of economic evaluations of DHIs as well as generic *quality* aspects such as *study design*.

### Suggestions for Further Research

This review provides a critique of methodological challenges that may arise in economic evaluations of DHIs tailored to pediatric and adolescent populations. Numerous DHIs are produced and adopted at pace, and economic evaluations considering the specific features of DHIs are needed. Economic evaluations with significant limitations may lead to suboptimal decisions and poor use of limited health care resources. In this section, we highlight some methodological areas that, in our view, would constitute interesting avenues for further research.

First, a common issue highlighted across the reviewed studies was the use of health outcome measures that are clinically relevant but less likely to meet the requirements for cost-effectiveness assessments, such as generic QoL measures. The development and validation of generic QoL measures for children and adolescents is beginning to emerge [52], but further research is warranted. For example, it is important to understand whether the measures are consistent across different age groups. Second, a broader perspective capturing indirect and non–health care costs and benefits is generally a preferred perspective in economic evaluations, but our review shows that it has not yet been used consistently for the evaluation of DHIs for children and adolescents. This could be addressed through more carefully designed data collection of indirect costs that account for health- and non–health-related burden to both children and their caregivers [11]. Third, although there have been some efforts to measure user participation (eg, user time) in the evaluation of DHIs [2,49,53], further research is needed to develop a more holistic value framework for formally valuing and incorporating user involvement in the cost and outcome analysis. In addition, following international guidelines and recommendations may improve the quality and consistency of economic evaluations of DHIs.

### Conclusions

Our study found that most (18/22, 82%) DHIs for children and adolescents are cost-effective or cost saving compared with the nondigital standard of care from either the health system/payer or societal viewpoint. However, there was a substantial degree of variability in cost-effectiveness results depending on the type of DHI, health care setting, and inclusion of certain input parameters in the economic evaluations. The cost-effectiveness of these DHIs appeared to be sensitive to the inclusion of certain cost components (particularly DHI implementation and supervision/training of health care professionals), intervention effect size, and its potential to be scaled up. This study highlights common methodological challenges directly related to the conduct of economic evaluations of DHIs. These included failure to measure user involvement, generic QoL outcomes, and indirect effects of DHIs, highlighting areas where further methodological research is required.

## Appendix

Multimedia Appendix 1 PRISMA (Preferred Reporting Items for Systematic Reviews and Meta-Analyses) checklist [15].

## Abbreviations

CBT: cognitive behavioral therapy
CHEERS: Consolidated Health Economic Evaluation Reporting Standards
CUA: cost-utility analysis
DALY: disability-adjusted life year
DHI: digital health intervention
ICER: incremental cost-effectiveness ratio
IPD: individual patient data
MNCH: maternal, newborn, and child health
NICE: National Institute for Health and Care Excellence
PRISMA: Preferred Reporting Items for Systematic Reviews and Meta-Analyses
QALY: quality-adjusted life year
QoL: quality of life

## Data Availability

Data sharing is not applicable to this article as no data sets were generated or analyzed during this study.

## References

[1] Murray E, Hekler EB, Andersson G, Collins LM, Doherty A, Hollis C, Rivera DE, West R, Wyatt JC (2016). Evaluating digital health interventions: key questions and approaches. Am J Prev Med.

[2] Gomes M, Murray E, Raftery J (2022). Economic evaluation of digital health interventions: methodological issues and recommendations for practice. Pharmacoeconomics.

[3] (2019). WHO guideline: recommendations on digital interventions for health system strengthening. World Health Organization.

[4] Soobiah C, Cooper M, Kishimoto V, Bhatia RS, Scott T, Maloney S, Larsen D, Wijeysundera HC, Zelmer J, Gray CS, Desveaux L (2020). Identifying optimal frameworks to implement or evaluate digital health interventions: a scoping review protocol. BMJ Open.

[5] Jiang X, Ming W-K, You JH (2019). The cost-effectiveness of digital health interventions on the management of cardiovascular diseases: systematic review. J Med Internet Res.

[6] Sanyal C, Stolee P, Juzwishin D, Husereau D (2018). Economic evaluations of eHealth technologies: a systematic review. PLoS One.

[7] Darling KE, Sato AF (2017). Systematic review and meta-analysis examining the effectiveness of mobile health technologies in using self-monitoring for pediatric weight management. Child Obes.

[8] Hollis C, Falconer CJ, Martin JL, Whittington C, Stockton S, Glazebrook C, Davies EB (2017). Annual research review: digital health interventions for children and young people with mental health problems - a systematic and meta-review. J Child Psychol Psychiatry.

[9] Rooksby M, Elouafkaoui P, Humphris G, Clarkson J, Freeman R (2015). Internet-assisted delivery of cognitive behavioural therapy (CBT) for childhood anxiety: systematic review and meta-analysis. J Anxiety Disord.

[10] Brigden A, Anderson E, Linney C, Morris R, Parslow R, Serafimova T, Smith L, Briggs E, Loades M, Crawley E (2020). Digital behavior change interventions for younger children with chronic health conditions: systematic review. J Med Internet Res.

[11] Gentili A, Failla G, Melnyk A, Puleo V, Tanna GL, Ricciardi W, Cascini F (2022). The cost-effectiveness of digital health interventions: a systematic review of the literature. Front Public Health.

[12] Hutchesson MJ, Rollo ME, Krukowski R, Ells L, Harvey J, Morgan PJ, Callister R, Plotnikoff R, Collins CE (2015). eHealth interventions for the prevention and treatment of overweight and obesity in adults: a systematic review with meta-analysis. Obes Rev.

[13] de la Torre-Díez I, López-Coronado M, Vaca C, Aguado JS, de Castro C (2015). Cost-utility and cost-effectiveness studies of telemedicine, electronic, and mobile health systems in the literature: a systematic review. Telemed J E Health.

[14] Mistry H (2012). Systematic review of studies of the cost-effectiveness of telemedicine and telecare. Changes in the economic evidence over twenty years. J Telemed Telecare.

[15] Moher D, Liberati A, Tetzlaff J, Altman DG, PRISMA Group (2009). Preferred reporting items for systematic reviews and meta-analyses: the PRISMA statement. PLoS Med.

[16] Bergmo TS (2015). How to measure costs and benefits of eHealth interventions: an overview of methods and frameworks. J Med Internet Res.

[17] Drummond M, Stoddart, GL, Torrance GW (1987). Methods for the Economic Evaluation of Health Care Programmes.

[18] van IJzendoorn MH, Bakermans-Kranenburg MJ (2020). Problematic cost-utility analysis of interventions for behavior problems in children and adolescents. New Dir Child Adolesc Dev.

[19] (2020). Do child QALYs = adult QALYs? Five reasons why they might not. London Office of Health Economics.

[20] Sanders GD, Neumann PJ, Basu A, Brock DW, Feeny D, Krahn M, Kuntz KM, Meltzer DO, Owens DK, Prosser LA, Salomon JA, Sculpher MJ, Trikalinos TA, Russell LB, Siegel JE, Ganiats TG (2016). Recommendations for conduct, methodological practices, and reporting of cost-effectiveness analyses: second panel on cost-effectiveness in health and medicine. JAMA.

[21] (2020). HMRC exchange rates for 2021: monthly. United Kingdom Government.

[22] US dollar (USD). European Central Bank.

[23] Australian Taxation Office (2022). Foreign currency exchange rates for the calendar year ending December 2021. Foreign currency equivalent to $1 Aust. Australian Taxation Office.

[24] Yang NH, Dharmar M, Yoo B-K, Leigh JP, Kuppermann N, Romano PS, Nesbitt TS, Marcin JP (2015). Economic evaluation of pediatric telemedicine consultations to rural emergency departments. Med Decis Making.

[25] Chatterton ML, Rapee RM, Catchpool M, Lyneham HJ, Wuthrich V, Hudson JL, Kangas M, Mihalopoulos C (2019). Economic evaluation of stepped care for the management of childhood anxiety disorders: results from a randomised trial. Aust N Z J Psychiatry.

[26] Olthuis JV, McGrath PJ, Cunningham CE, Boyle MH, Lingley-Pottie P, Reid GJ, Bagnell A, Lipman EL, Turner K, Corkum P, Stewart SH, Berrigan P, Sdao-Jarvie K (2018). Distance-delivered parent training for childhood disruptive behavior (Strongest Families™): a randomized controlled trial and economic analysis. J Abnorm Child Psychol.

[27] LeFevre A, Cabrera-Escobar MA, Mohan D, Eriksen J, Rogers D, Neo Parsons A, Barre I, Jo Y, Labrique A, Coleman J (2018). Forecasting the value for money of mobile maternal health information messages on improving utilization of maternal and child health services in Gauteng, South Africa: cost-effectiveness analysis. JMIR Mhealth Uhealth.

[28] Jo Y, LeFevre A, Ali H, Mehra S, Alland K, Shaikh S, Haque R, Pak ES, Chowdhury M, Labrique AB (2021). mCARE, a digital health intervention package on pregnancy surveillance and care-seeking reminders from 2018 to 2027 in Bangladesh: a model-based cost-effectiveness analysis. BMJ Open.

[29] Willcox M, Moorthy A, Mohan D, Romano K, Hutchful D, Mehl G, Labrique A, LeFevre A (2019). Mobile technology for community health in ghana: is maternal messaging and provider use of technology cost-effective in improving maternal and child health outcomes at scale?. J Med Internet Res.

[30] Kawakatsu Y, Oyeniyi Adesina A, Kadoi N, Aiga H (2020). Cost-effectiveness of SMS appointment reminders in increasing vaccination uptake in Lagos, Nigeria: a multi-centered randomized controlled trial. Vaccine.

[31] Jo Y, LeFevre AE, Healy K, Singh N, Alland K, Mehra S, Ali H, Shaikh S, Haque R, Christian P, Labrique AB (2019). Costs and cost-effectiveness analyses of mCARE strategies for promoting care seeking of maternal and newborn health services in rural Bangladesh. PLoS One.

[32] Zurovac D, Larson BA, Sudoi RK, Snow RW (2012). Costs and cost-effectiveness of a mobile phone text-message reminder programmes to improve health workers' adherence to malaria guidelines in Kenya. PLoS One.

[33] Modi D, Saha S, Vaghela P, Dave K, Anand A, Desai S, Shah P (2020). Costing and cost-effectiveness of a mobile health intervention (ImTeCHO) in improving infant mortality in tribal areas of Gujarat, India: cluster randomized controlled trial. JMIR Mhealth Uhealth.

[34] Bowser DM, Shepard DS, Nandakumar A, Okunogbe A, Morrill T, Halasa-Rappell Y, Jordan M, Mushi F, Boyce C, Erhunmwunse OA (2018). Cost effectiveness of mobile health for Antenatal care and facility births in Nigeria. Ann Glob Health.

[35] Prinja S, Bahuguna P, Gupta A, Nimesh R, Gupta M, Thakur JS (2018). Cost effectiveness of mHealth intervention by community health workers for reducing maternal and newborn mortality in rural Uttar Pradesh, India. Cost Eff Resour Alloc.

[36] Jolstedt M, Wahlund T, Lenhard F, Ljótsson B, Mataix-Cols D, Nord M, Öst L-G, Högström J, Serlachius E, Vigerland S (2018). Efficacy and cost-effectiveness of therapist-guided internet cognitive behavioural therapy for paediatric anxiety disorders: a single-centre, single-blind, randomised controlled trial. Lancet Child Adolesc Health.

[37] De Bruin EJ, van Steensel FJ, Meijer AM (2016). Cost-effectiveness of group and internet cognitive behavioral therapy for insomnia in adolescents: results from a randomized controlled trial. Sleep.

[38] Lalouni M, Ljótsson B, Bonnert M, Ssegonja R, Benninga M, Bjureberg J, Högström J, Sahlin H, Simrén M, Feldman I, Hedman-Lagerlöf E, Serlachius E, Olén O (2019). Clinical and cost effectiveness of online cognitive behavioral therapy in children with functional abdominal pain disorders. Clin Gastroenterol Hepatol.

[39] Lenhard F, Ssegonja R, Andersson E, Feldman I, Rück C, Mataix-Cols D, Serlachius E (2017). Cost-effectiveness of therapist-guided internet-delivered cognitive behaviour therapy for paediatric obsessive-compulsive disorder: results from a randomised controlled trial. BMJ Open.

[40] Nordh M, Wahlund T, Jolstedt M, Sahlin H, Bjureberg J, Ahlen J, Lalouni M, Salomonsson S, Vigerland S, Lavner M, Öst L-G, Lenhard F, Hesser H, Mataix-Cols D, Högström J, Serlachius E (2021). Therapist-guided internet-delivered cognitive behavioral therapy vs internet-delivered supportive therapy for children and adolescents with social anxiety disorder: a randomized clinical trial. JAMA Psychiatry.

[41] Aspvall K, Sampaio F, Lenhard F, Melin K, Norlin L, Serlachius E, Mataix-Cols D, Andersson E (2021). Cost-effectiveness of internet-delivered vs in-person cognitive behavioral therapy for children and adolescents with obsessive-compulsive disorder. JAMA Netw Open.

[42] Lee YY, Barendregt JJ, Stockings EA, Ferrari AJ, Whiteford HA, Patton GA, Mihalopoulos C (2017). The population cost-effectiveness of delivering universal and indicated school-based interventions to prevent the onset of major depression among youth in Australia. Epidemiol Psychiatr Sci.

[43] Sampaio F, Bonnert M, Olén O, Hedman E, Lalouni M, Lenhard F, Ljótsson B, Ssegonja R, Serlachius E, Feldman I (2019). Cost-effectiveness of internet-delivered cognitive-behavioural therapy for adolescents with irritable bowel syndrome. BMJ Open.

[44] Wasil A, Kacmarek C, Osborn T, Palermo EH, DeRubeis RJ, Weisz JR, Yates BT (2021). Economic evaluation of an online single-session intervention for depression in Kenyan adolescents. J Consult Clin Psychol.

[45] van den Wijngaart LS, Kievit W, Roukema J, Boehmer AL, Brouwer ML, Hugen CA, Niers LE, Sprij AJ, Rikkers-Mutsaerts ER, Rottier BL, Verhaak CM, Pijnenburg MW, Merkus PJ (2017). Online asthma management for children is cost-effective. Eur Respir J.

[46] Hedman E, Ljótsson B, Lindefors N (2012). Cognitive behavior therapy via the internet: a systematic review of applications, clinical efficacy and cost-effectiveness. Expert Rev Pharmacoecon Outcomes Res.

[47] Arnberg FK, Linton SJ, Hultcrantz M, Heintz E, Jonsson U (2014). Internet-delivered psychological treatments for mood and anxiety disorders: a systematic review of their efficacy, safety, and cost-effectiveness. PLoS One.

[48] (2018). Evidence standards framework for digital health technologies. National Institute for Health and Care Excellence.

[49] Babigumira JB, Dolan S, Shade S, Puttkammer N, Bale J, Tolentino H, Santas XM (2021). Applied economic evaluation of digital health interventions. Centers for Disease Control and Prevention, International Training & Education Center for Health.

[50] Puleo V, Gentili A, Failla G, Melnyk A, Di Tanna G, Ricciardi W, Cascini F (2021). Digital health technologies: a systematic review of their cost-effectiveness. Eur J Public Health.

[51] Husereau D, Drummond M, Augustovski F, de Bekker-Grob E, Briggs AH, Carswell C, Caulley L, Chaiyakunapruk N, Greenberg D, Loder E, Mauskopf J, Mullins CD, Petrou S, Pwu R-F, Staniszewska S, CHEERS 2022 ISPOR Good Research Practices Task Force (2022). Consolidated Health Economic Evaluation Reporting Standards 2022 (CHEERS 2022) statement: updated reporting guidance for health economic evaluations. Value Health.

[52] Rowen D, Rivero-Arias O, Devlin N, Ratcliffe J (2020). Review of valuation methods of preference-based measures of health for economic evaluation in child and adolescent populations: where are we now and where are we going?. Pharmacoeconomics.

[53] Pagliari C (2007). Design and evaluation in eHealth: challenges and implications for an interdisciplinary field. J Med Internet Res.

